# *Acinetobacter* spp. bloodstream infection in hematological patients: a 10-year single-center study

**DOI:** 10.1186/s12879-023-08789-6

**Published:** 2023-11-14

**Authors:** Jia Li, Xiaomeng Feng, Jieru Wang, Qingsong Lin, Yizhou Zheng, Fengkui Zhang, Yingchang Mi, Xiaofan Zhu, Erlie Jiang, Zhijian Xiao, Jianxiang Wang, Sizhou Feng

**Affiliations:** 1 State Key Laboratory of Experimental Hematology, National Clinical Research Center for Blood Diseases, Haihe Laboratory of Cell Ecosystem, Institute of Hematology & Blood Diseases Hospital, Chinese Academy of Medical Sciences & Peking Union Medical College, No. 288 Nanjing Road, Tianjin, 300020 China; 2 Tianjin Institutes of Health Science, Tianjin, 301600 China

**Keywords:** *Acinetobacter*, Bloodstream Infection, Risk factors, Treatment, Outcome

## Abstract

**Purpose:**

This study investigated the clinical and antimicrobial characteristics of *Acinetobacter* spp. bloodstream infection (BSI) in hematological patients. Risk factors for 30-day mortality and carbapenem-resistant *Acinetobacter* spp. (CRA) BSI acquisition were also identified.

**Methods:**

We reviewed forty hematological patients with *Acinetobacter* spp. BSI in a large Chinese blood disease hospital between 2013 and 2022. The remaining CRA isolates were subjected to whole-genome sequencing.

**Results:**

The 30-day mortality rate was high at 35%. Hematological patients with *Acinetobacter* spp. BSI often presented with severe conditions and co-infections at multiple sites. All strains were colistin-susceptible and 40.0% were CR. Multivariate analysis identified several risk factors associated with CRA BSI acquisition, including previous exposure to carbapenems within 30 days and CRA colonization. Very severe aplastic anaemia, tetracycline-resistant *Acinetobacter* spp. BSI, and unresolved neutropenia after infection were closely associated with 30-day mortality. Non-survivors often presented with higher median PCT and CRP levels and severe complications, such as intracranial infection, cardiac dysfunction, respiratory failure, and severe sepsis or septic shock. Our study also identified inappropriate empirical antibiotic therapy as an independent predictor of 30-day mortality (OR: 11.234, 95% CI: 1.261–20.086, P = 0.030). This study was the first to report *A. oleivorans* as a human pathogen, and to identify its unique oxacillinase, OXA-325.

**Conclusion:**

An environment-originated non-pathogenic species can become pathogenic when the body’s immunity is compromised. Our results also highlighted the importance of improving neutropenia after infection, treating severe organ dysfunction, and administering appropriate empirical antibiotic therapy to reduce mortality in this patient population.

**Supplementary Information:**

The online version contains supplementary material available at 10.1186/s12879-023-08789-6.

## Introduction

*Acinetobacter* spp. is a complex genus that commonly causes nosocomial infections, predominantly pneumonia and bloodstream infection (BSI) [1]. While *A. baumannii* remains one of the most significant pathogens responsible for *Acinetobacter* spp. BSI [2–5], there has been a recent emergence of other species, including *A. pittii, A. calcoaccius, A. lwoffii, A. junii, A. soli*, and *A. nosocomialis*, as common pathogens causing nosocomial infections [6, 7]. Several studies have suggested that the severity of infections caused by non-*baumannii A. calcoaceticus-baumannii* (Acb) complex was comparable to that of infections caused by *A. baumannii*, rather than being less severe [8–11].

Carbapenems are commonly used to treat serious infections caused by *Acinetobacter baumannii (A baumannii).* However, long-term antimicrobial resistance surveillance of nosocomial gram-negative bacilli in China from 2010 to 2018 demonstrated that the resistance rate of *A. baumannii* to carbapenem drugs is gradually increasing [12]. Resistance to carbapenems has garnered significant attention due to its serious threat to public health [4, 13, 14]. However, the risk factors for developing carbapenem-resistant *Acinetobacter* (CRA) BSI have not been extensively reported.

The prevalence of *Acinetobacter* spp. BSI is relatively low in patients with hematological disorders, but it poses a significant clinical challenge due to limited treatment options and high mortality rates. In a multicenter study in China, *A. baumannii* bacteremia accounted for only 2.9% (40/1358) of all cases of bacteremia [15]. In our center, the 30-day mortality rates for hematological patients with *Aeromonas* bacteremia [16], multidrug-resistant (MDR) *Pseudomonas aeruginosa* bacteremia [17], CR *Enterobacteriaceae* bacteremia [13], and *Stenotrophomonas maltophilia* bacteremia (unpublished data) were 15.9%, 8.5%, 34%, and 29.3%, respectively. In contrast, the 30-day mortality rate of *Acinetobacter* spp. BSI ranges from 33–72% [2–5]. Data regarding outcomes in hematological patients with *Acinetobacter* spp. BSI remain limited [14, 15]. Some articles [14, 15] have emphasized the impact of appropriate antibiotic therapy within 48 h, carbapenem resistance, and higher infection severity score on the prognosis of CR *A. baumannii* BSI. However, further studies are needed to comprehensively analyze the risk factors for mortality in hematological patients with *Acinetobacter* spp. BSI and to implement infection control measures to improve prognosis.

Therefore, this retrospective study of hematological patients with *Acinetobacter* spp. BSI aimed to (i) investigate the clinical characteristics, the antibiotic resistance profiles and genomospecies of isolated strains, (ii) evaluate the prognostic factors of *Acinetobacter* spp. BSI, and (iii) identify risk factors for CRA BSI acquisition.

## Patients and methods

### Data collection

This study retrospectively analysed 40 patients diagnosed with hematological diseases and *Acinetobacter* spp. BSI between April 2013 and July 2022 at a 766-bed tertiary blood disease hospital in Tianjin, China. Data collected from medical records included age, sex, diagnosis, clinical manifestations, medical history, microbiological test results, treatment, and outcomes. This study was approved by the Ethics Committee of the Institute of Hematology and Blood Diseases Hospital.

### Definitions

The onset of BSI was defined as the collection date of positive blood culture samples. Laboratory examinations, such as routine blood and biochemical tests, were performed within 24 h [18]. Neutropenia was defined as an absolute neutrophil count < 0.5 × 10^9^ cells/L. Unresolved neutropenia was defined as neutropenia for > 14 consecutive days before or after infection or until death. Administration of any antibiotic for > = 48 h within the month before the onset of BSI was regarded as previous antibiotic use. Empirical antibiotic therapy was defined as any antibiotic administered to febrile patients suspected of having bacteremia before the susceptibility results were available. Appropriate empirical antibiotic therapy was defined as the administration of one or more active agents against *Acinetobacter* spp. at an adequate dose within 24 h after the culture was obtained. The treatment administered after obtaining susceptibility results was defined as definitive therapy. Definitive therapy was stratified into monotherapy and combination therapy [17]. The following cut-off values were used for the primary analysis: 0.5 µg/L for procalcitonin (PCT), 10 mg/L for C-reactive protein (CRP), 104 µmol/L for creatine (Cr), and 50 U/L for alanine aminotransferase.

### Antimicrobial susceptibility testing

The specimens were collected and cultured according to the Clinical and Laboratory Standards Institute’s M100. An automated system (VITEK 2 Compact) was used to conduct the initial strain identification and antimicrobial susceptibility tests in the hospital’s microbiology laboratory using broth microdilution and disk diffusion methods. Following this, the strains were collected by means of filter paper and placed into strain storage tubes, where they were stored in a refrigerator at -80 °C.

### Bacterial strain and DNA extraction

Upon the resuscitation of the CRA strains maintained in our laboratory, a single colony was carefully selected. It was inoculated onto a blood agar medium and cultured at 37 °C with 5% CO2 for a duration of 20 h. Genomic DNA was subsequently extracted from cell pellets using a Bacteria DNA Kit (OMEGA) following the manufacturer’s instructions. The purified DNA samples were subjected to a rigorous quality control assessment, and only high-quality DNA samples (OD260/280 = 1.8 ~ 2.0, >6ug) were employed for the construction of fragment libraries.

### Illumina HiSeq sequencing, genome assembly and genome annotation

For Illumina paired-end sequencing, a minimum of 1 µg of genomic DNA was utilized. Paired-end libraries with 400 bp insert sizes were meticulously prepared according to Illumina’s standard protocol. The process involved fragmenting the purified genomic DNA, generating blunt ends, ligating adapters, and subsequently purifying, enriching, and PCR amplifying the fragments. The qualified Illumina paired-end library was used for Illumina NovaSeq 6000 sequencing (150 bp*2). The raw paired-end reads underwent a thorough trimming and quality control process using Trimmomatic. The clean data resulting from these quality control procedures were employed for subsequent analysis. Genome assembly was meticulously executed using ABySS with multiple-Kmer parameters, and the GapCloser software was used to fill remaining gaps and correct base polymorphisms. The Whole Genome Sequencing (WGS) results allowed us to reclassify the CRA strains based on the Genome Taxonomy Database (GTDB) [19]. Sequence alignment and gene annotation were performed using a comprehensive antimicrobial resistance database (https://card.mcmaster.ca/) and a virulence factor database (http://www.mgc.ac.cn/VFs/main.htm). This whole-genome shotgun project was deposited in GenBank under the accession number **PRJNA883531**. Shanghai Biozeron Biotechnology Co., Ltd performed sequencing.

### Statistical analysis

SPSS software (version 24.0; Chicago, IL, USA) was used to analyse the data. Categorical variables were compared using the chi-squared or Fisher’s exact tests. Continuous variables were expressed as the median and interquartile range (IQR), and differences were identified using the two-sample t-test or Mann-Whitney U test. The Kaplan-Meier method was used to plot survival curves (log-rank test). In summary, for variables with a significance level of P < 0.05 in the single-factor analysis, we selected one baseline variable that is most clinically relevant in patients’ general condition, microbial characteristics, and antibiotic interventions, respectively, for subsequent multifactorial analysis. A logistic regression model was used to evaluate potential risk factors in multivariate analyses. Statistical significance was set at P-values < 0.05 [20].

## Results

### Clinical characteristics and outcomes

Complete clinical data were obtained from 40 patients with *Acinetobacter* spp. BSI during the study period. The clinical characteristics of patients with *Acinetobacter* spp. BSI who survived or died within 30 days are summarised in Table 1. The sex distribution was similar between the groups, with a median age of 25.5 years (range: 1–62 years). The underlying diseases included very severe aplastic anaemia (VSAA), acute lymphoblastic leukaemia, acute myeloid leukaemia, myelodysplastic syndrome, and diffuse large B-cell lymphoma. During the same period, CRA colonization occurred in 25% (n = 10) of the patients with *Acinetobacter* spp. BSI. All patients developed fever, and 55.0% (n = 22) were diagnosed with pneumonia. 30% of the patients (n = 12) presented with pleural effusion (50.0% vs. 19.2%, P = 0.071), which accounted for a marginally higher proportion of non-survivors; 11 presented with oral mucositis or pharyngitis; two presented with sinus infections; four presented with skin or soft tissue infections; and six presented with perianal infections. In addition, five and nine patients developed an intracranial infection and respiratory failure, respectively. 20% (n = 10) of the patients with *Acinetobacter* spp. BSI eventually developed severe sepsis or septic shock.

**Table 1:** Characteristics of patients with* Acinetobacter* spp. bloodstream infection, stratified by outcome

				Univariate analysis	Multivariate analysis	
Characteristics	Total n = 40	Survivors n = 26	Non-survivors n = 14	P value	OR (95% CI)	P value
**Male sex**	22 (55.0)	14 (53.8)	8 (57.1)	0.842		
**Age**						
median (range)	25.5 (1–62)	18.5 (7–40)	31 (17–49)	0.243		
≥ 45	11 (27.5)	6 (23.1)	5 (35.7)	0.469		
**Underlying diseases**						
VSAA	9 (22.5)	3 (11.5)	6 (42.9)	0.044*		
ALL	11 (27.5)	10 (38.5)	1 (7.1)	***0.061***		
AML	17 (42.5)	10 (38.5)	7 (50.0)	0.481		
MDS	2 (5.0)	2 (7.7)	0 (0.0)	0.533		
DLBCL	1 (2.5)	1 (3.8)	0 (0.0)	1.000		
**Diabetes**	3 (7.5)	2 (7.7)	1 (7.1)	1.000		
**Carbapenem-resistant** ***Acinetobacter*** **spp. colonization**	10 (25.0)	6 (23.1)	4 (28.6)	0.718		
**Total length of hospital stay**	35 (23–59)	35 (25–58)	35 (22–69)	0.790		
**Length of hospital stay before BSI**	15 (5–24)	12 (5–19)	21 (8–36)	0.118		
**Accompanying symptoms**						
Fever	40 (100.0)	26 (100.0)	14 (100.0)			
Oral mucositis or pharyngitis	11 (27.5)	6 (23.1)	5 (35.7)	0.469		
Sinus infection	2 (5.0)	1 (3.8)	1 (7.1)	1.000		
Pneumonia	22 (55.0)	12 (46.2)	10 (71.4)	0.125		
Pleural effusion	12 (30.0)	5 (19.2)	7 (50.0)	***0.071***		
Intracranial infection	5 (12.5)	0 (0.0)	5 (35.7)	0.003*		
Skin or soft tissue infection	4 (10.0)	1 (3.8)	3 (21.4)	0.115		
Urinary tract or perineal infection	0 (0.0)	0 (0.0)	0 (0.0)			
Perianal infection	6 (15.0)	4 (15.4)	2 (14.3)	1.000		
Respiratory failure	9 (22.5)	1 (3.8)	8 (57.1)	< 0.001*		
Cardiac dysfunction	13 (32.5)	5 (19.2)	8 (57.1)	0.031*	0.776 (0.068–8.912)	0.838
Severe sepsis or septic shock	10 (25.0)	1 (3.8)	9 (64.3)	< 0.001*		
**Laboratory results**						
Neutropenia at the onset of BSI	31 (77.5)	17 (65.4)	14 (100.0)	0.016*		
Unresolved neutropenia before infection	13 (35.1) ^a^	6 (24.0) ^a^	7 (58.3) ^a^	***0.067***		
Unresolved neutropenia after infection	21 (52.5)	8 (30.8)	13 (92.9)	0.001*		
Hypoproteinemia	19 (48.7) ^b^	10 (40.0) ^b^	9 (64.3) ^b^	0.146		
PCT (ug/L)	0.81 (0.11–2.33)	0.14 (0.10–1.10)	1.79 (0.53–21.60)	0.025*		
CRP (mg/L)	37.16 (11.07-117.41)	12.40 (9.32–58.30)	128.13 (61.00-243.78)	0.001*		
**Antibiotic use**						
Previous antibiotic use	34 (85.0)	21 (80.8)	13 (92.9)	0.399		
Antimicrobial exposure at the onset of BSI	21 (52.5)	12 (46.2)	9 (64.3)	0.273		
Inappropriate empirical therapy	12 (30.0)	4 (15.4)	8 (57.1)	0.011*	11.234 (1.261–20.086)	0.030*
Definitive combination therapy	21 (52.5)	13 (50.0)	8 (57.1)	0.666		
Antifungal agents in combination	28 (70.0)	17 (65.4)	11 (78.6)	0.484		
Time of antibiotic treatment after BSI	14 (6–25)	16 (8–32)	5 (4–13)	0.006*		
**Resistance profiles**						
Carbapenem-resistant	16 (40.0)	9 (34.6)	7 (50.0)	0.343		
Multidrug-resistant	19 (47.5)	11 (42.3)	8 (57.1)	0.370		
Extensively drug-resistant	5 (12.5)	1 (3.8)	4 (28.6)	0.043*		
Tetracycline-resistant	8 (25.8) ^c^	3 (13.6) ^c^	5 (55.6) ^c^	0.027*	3.568 (0.341–37.313)	0.288
Broad-spectrum cephalosporins-resistant	37 (92.5)	24 (92.3)	13 (92.9)	1.000		
Fluoroquinolones-resistant	13 (32.5) ^d^	7 (28.0) ^d^	6 (42.9) ^d^	0.482		
Aminoglycosides-resistant	14 (35.0)	7 (26.9)	7 (50.0)	0.178		

*Note*: Categorical variables are presented as numbers (percentiles); continuous variables are presented as median (interquartile range, IQR) unless otherwise stated. A P value in italics and bold means < 0.1, followed by * means < 0.05.

*Abbreviations*: OR, odds ratio; CI, confidence interval; VSAA, very severe aplastic anemia; ALL, acute lymphoblastic leukemia; AML, acute myeloid leukemia; MDS, myelodysplastic syndrome; DLCBL, diffuse large B-cell lymphoma; PCT, procalcitonin; CRP, C-reactive protein; Cr, creatine; ALT, alanine aminotransferase. BSI, bloodstream infection. ^a^37, 25 and 12 evaluable patients. ^b^39, 25 and 14 evaluable patients. ^c^31, 22 and 9 evaluable patients. ^d^39, 25 and 14 evaluable patients

The median length of hospital stay was 35 days. Non-survivors had a marginally longer median length of hospital stay (median: 21, [IQR: 8–36] days vs. median: 12, [IQR: 5–19] days; P = 0.118). Seven of the 40 patients died within 7 days, with a mortality rate of 25% at 14 days. By day 30, after the onset of bacteremia, treatment was ineffective in 37.5% (n = 15) of the patients. Furthermore, the 30-day all-cause mortality rate among patients with *Acinetobacter* spp. bloodstream infections (BSI) was as high as 35%. The 90-day mortality rate stood at 42.5%.

### Antimicrobial therapy

All patients received empirical antibiotic therapy immediately after collecting blood culture samples. None of the patients used antibiotics for prophylaxis. Previous antibiotic use (92.9% vs. 80.8%, P = 0.399) and antimicrobial exposure at the onset of BSI (64.3% vs. 46.2%, P = 0.273) were not associated with 30-day mortality (Table 1). Moreover, the proportion of patients receiving appropriate empirical antibiotics differed markedly between non-survivors and survivors (15.4% vs. 57.1%, P = 0.011). A total of 52.5% (n = 21) of patients received definitive combination therapy, with no noticeable effect on 30-day survival (50.0% vs. 57.1%, P = 0.666).

The following data were analysed to understand the influence of appropriate empirical antibiotic therapy and combined target antibiotics on patients (Supplementary Table 1). Patients with CRA (91.7% vs. 17.9%, P < 0.001) and MDR *Acinetobacter* spp. (MDRA) (100.0% vs. 25.0%, P < 0.001) bacteremia often received inappropriate empirical antibiotic therapy within 24 h, and the definite therapy tended to be in combination (CRA: 61.9% vs. 15.8%, P = 0.003; MDRA: 66.7% vs. 26.3%, P = 0.011). Patients who received appropriate empirical antibiotic therapy had significantly higher microbial cure rates (57.1% vs. 8.3%, P = 0.004) and lower mortality rates (21.4% vs. 66.7%, P = 0.011) within 30 days.

### Antimicrobial resistance of the isolates

**Fig. 1:**
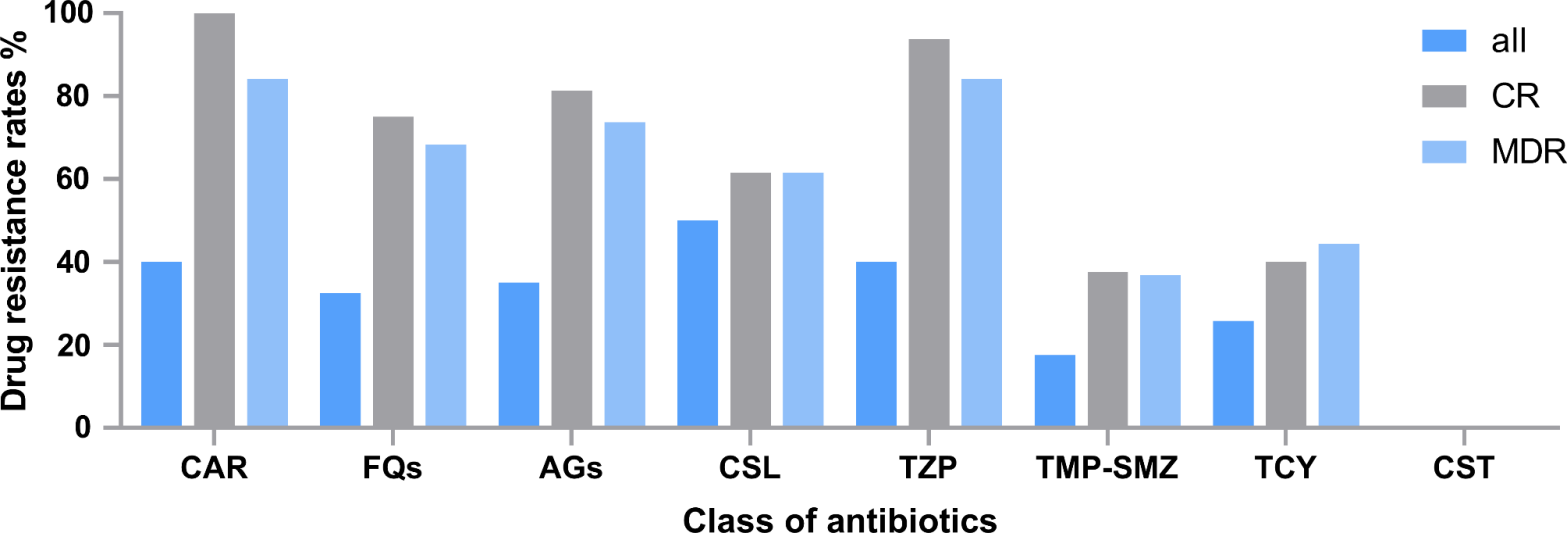
Distribution of drug resistance according to carbapenem-resistant (CR) and multidrug-resistant (MDR) stratifications. CAR, carbapenems; FQs, fluoroquinolones; AGs, aminoglycosides; CSL, cefoperazone-sulbactam; TZP, piperacillin-tazobactam; TMP-SMZ, trimethoprim-sulfamethoxazole; TCY, tetracycline; CST, colistin

Antimicrobial resistance of *Acinetobacter* spp. isolates from the bloodstream is shown in Table 2. Of the 40 isolated strains, 40% (n = 16) were CRA, 47.5% (n = 19) were MDRA, and 25.8% (n = 8) were tetracycline-resistant *Acinetobacter* spp. Colistin exhibited the highest antimicrobial activity against the isolated strains (100.0%), followed by minocycline (92.3%), doxycycline (90.0%), trimethoprim-sulfamethoxazole (TMP-SMZ) (82.5%) and tigecycline (80.6%). Figure 1 shows the drug resistance of *Acinetobacter* spp. isolates to eight classes of antibiotics according to CR and MDR stratifications. The rates of resistance to carbapenems, fluoroquinolones, aminoglycosides, cefoperazone-sulbactam, piperacillin-tazobactam, TMP-SMZ, tetracyclines, and colistin of the CR strains were 100.0%, 75.0%, 81.3%, 61.5%, 93.8%, 37.5%, 40.0%, and 0.0%, respectively. In contrast, those of MDR strains were 84.2%, 68.4%, 73.7%, 61.5%, 84.2%, 36.8%, 44.4%, and 0.0%, respectively. For tetracycline-resistant *Acinetobacter* spp. BSI, TMP-SMZ and colistin were available. Furthermore, we have included the Minimum Inhibitory Concentrations for all 40 strains in tabular format (Supplementary Table 2).

**Table 2:** Clinical characteristics and risk factors associated with bloodstream infection caused by carbapenem-resistant* Acinetobacter *spp.

			Univariate analysis	Multivariate analysis	
Characteristics	CSA n = 24	CRA n = 16	P value	OR (95% CI)	P value
**Male sex**	11 (45.8)	11 (68.8)	0.154		
**Age**					
median (range)	16 (1–62)	27.5 (1–54)	0.404		
≥ 45	6 (25.0)	5 (31.3)	0.728		
**Underlying diseases**					
VSAA	3 (12.5)	6 (37.5)	0.120		
ALL	9 (37.5)	2 (12.5)	0.148		
AML	10 (41.7)	7 (43.8)	0.896		
MDS	1 (4.2)	1 (6.3)	1.000		
DCLCB	1 (4.2)	0 (0.0)	1.000		
**Diabetes**	1 (4.2)	2 (12.5)	0.553		
**Accompanying symptoms**					
Respiratory failure	3 (12.5)	6 (37.5)	0.120		
Cardiac dysfunction	5 (20.8)	8 (50.0)	***0.054***		
Severe sepsis/septic shock	4 (16.7)	6 (37.5)	0.159		
Hypoproteinemia	8 (34.8) ^a^	11 (68.8) ^a^	0.037*	5.708 (0.823–39.566)	***0.078***
**Carbapenem-resistant** ***Acinetobacter*** **spp. colonization**	0 (0.0%)	10 (62.5)	< 0.001*	11.949 (1.799–79.363)	0.010*
**Length of hospital stay before BSI**	16 (4–28)	14 (5–24)	0.723		
**Interventions within 30 days**					
Central venous catheterization	22 (91.7)	12 (75.0)	0.195		
Intravenous glucocorticoid	14 (58.3)	11 (68.8)	0.505		
Immunosuppressive therapy	21 (87.5)	12 (75.0)	0.407		
Chemotherapy	17 (70.8)	8 (50.0)	0.182		
ATG/ALG	1 (4.2)	2 (12.5)	0.553		
HSCT	0 (0.0)	2 (12.5)	0.154		
**Laboratory results**					
Unresolved neutropenia before infection	8 (36.4) ^b^	5 (33.3) ^b^	0.850		
Unresolved neutropenia after infection	11 (45.8)	10 (62.5)	0.301		
Time of antibiotic treatment after BSI	11 (5–16)	20.5 (6–40)	0.220		
**Antimicrobial exposure at the onset of BSI**	8 (33.3)	13 (81.3)	0.003*		
**Previous antibiotic use**	18 (75.0)	16 (100.0)	***0.064***		
BLICs	9 (37.5)	9 (56.3)	0.243		
Broad-spectrum cephalosporins	1 (4.2)	4 (25.0)	0.138		
Carbapenems	5 (20.8)	12 (75.0)	0.001*	6.927 (1.125–42.638)	0.037*
Tigecycline	1 (4.2)	2 (12.5)	0.553		
Trimethoprim-sulfamethoxazole	4 (16.7)	1 (6.3)	0.631		
**Multidrug-resistant**	3 (12.5)	16 (100.0)	< 0.001*		
**Inappropriate empirical therapy**	1 (4.2)	11 (68.8)	< 0.001*		

*Note*: Categorical variables are presented as numbers (percentiles); continuous variables are presented as median (interquartile range, IQR) unless otherwise stated. A P value in italics and bold means < 0.1, followed by * means < 0.05.

*Abbreviations*: CSA, carbapenem-susceptible *Acinetobacter* spp.; CRA, carbapenem-resistant *Acinetobacter* spp.; OR, odds ratio; CI, confidence interval; VSAA, very severe aplastic anemia; ALL, acute lymphoblastic leukemia; AML, acute myeloid leukemia; MDS myelodysplastic syndrome; DLCBL, diffuse large B-cell lymphoma; BSI, bloodstream infection; BLICs, β-lactam/β-lactamase inhibitor combinations. ^a^39, 23 and 16 evaluable patients. ^b^37, 22 and 15 evaluable patients

### Risk factors for CRA BSI acquisition

In the univariate analysis, patients with hypoproteinaemia (68.8% vs. 34.8%, P = 0.037) and those with antimicrobial exposure at the onset of BSI (81.3% vs. 33.3%, P = 0.003) had an increased risk of developing CRA BSI **(**Table 3**)**. Similarly, CRA colonization (62.5% vs. 0.0%, P < 0.001) and history of carbapenem use within 30 days (75.0% vs. 20.8%, P = 0.001) were factors associated with CRA BSI. In addition, a marginally larger proportion of patients had cardiac dysfunction (50.0% vs. 20.8%, P = 0.054). In the multivariate analysis, CRA colonization (Odds ratio (OR): 11.949, 95% CI: 1.799–79.363, P = 0.010) and previous exposure to carbapenems within 30 days (OR: 6.927, 95% CI: 1.125–42.638, P = 0.037) were independent risk factors for CRA BSI.

**Table 3:** Drug resistance of common antibiotics in primary *Acinetobacter* spp. bloodstream infection

Antibiotics	Resistant rate (%)
Piperacillin	45.0%
Ciprofloxacin	30.0%
Levofloxacin	27.5%
Moxifloxacin	30.0%
Tobramycin	27.5%
Gentamicin	36.8%
Cefotaxime	83.9%
Ceftazidime	52.5%
Ceftriaxone	97.4%
Cefepime	37.5%
Trimethoprim-sulfamethoxazole	17.5%
Ampicillin-sulbactam	34.2%
Piperacillin-tazobactam	40.0%
Cefoperazone-sulbactam	50.0%
Meropenem	46.2%
Imipenem	40.0%
Doripenem	48.4%
Tetracycline	19.4%
Minocycline	7.7%
Doxycycline	10.0%
Tigecycline	19.4%
Colistin	0.0%

Antimicrobial susceptibility was determined according to the Clinical and Laboratory Standards Institute (CLSI) criteria

### Risk factors for 30-day mortality in patients with *Acinetobacter* Spp. BSI

The prognostic factors associated with 30-day mortality were analysed after *Acinetobacter* spp. BSI with respect to hosts, pathogens, and treatments **(**Table 1**)**. In the underlying disease, patients with VSAA had a higher 30-day mortality rate (42.9% vs. 11.5%, P = 0.044). The patients who died within 30 days were more likely to have intracranial infections (35.7% vs. 0.0%, P = 0.003), respiratory failure (57.1% vs. 3.8%, P < 0.001), cardiac dysfunction (57.1% vs. 19.2%, P = 0.031), and severe sepsis/septic shock (64.3% vs. 3.8%, P < 0.001). Most hematological patients (77.5%, n = 31) had neutropenia at the onset of *Acinetobacter* spp. BSI. Patients who died within 30 days usually had unresolved neutropenia for > 14 consecutive days, either before (58.3% vs. 24.0%, P = 0.067) or after infection (92.9% vs. 30.8%, P = 0.001). The non-survivors had significantly higher median PCT (median: 1.79 [IQR: 0.53–21.60] ug/L vs. median: 0.14 [IQR: 0.10–1.10] ug/L, P = 0.025) and CRP (median: 128.13 [IQR: 61.00–243.78] mg/L vs. median: 12.40 [IQR: 9.32–58.30] mg/L, P = 0.001) levels. These results suggest that these biomarkers are useful tools for assessing disease severity. Surprisingly, neither CRA (50.0% vs. 34.6%, P = 0.343) nor MDRA (57.1% vs. 42.3%, P = 0.370) were risk factors for 30-day mortality. Extensively drug-resistant *Acinetobacter* spp. (XDRA) (28.6% vs. 3.8%, P = 0.043) and tetracycline-resistant *Acinetobacter* spp. (55.6% vs. 13.6%, P = 0.027) were predictors of poor prognosis. In the multivariate model, inappropriate empirical antibiotic therapy was an independent risk factor for 30-day mortality (OR: 11.234, 95% CI: 1.261–20.086, P = 0.030).

**Fig. 2:**
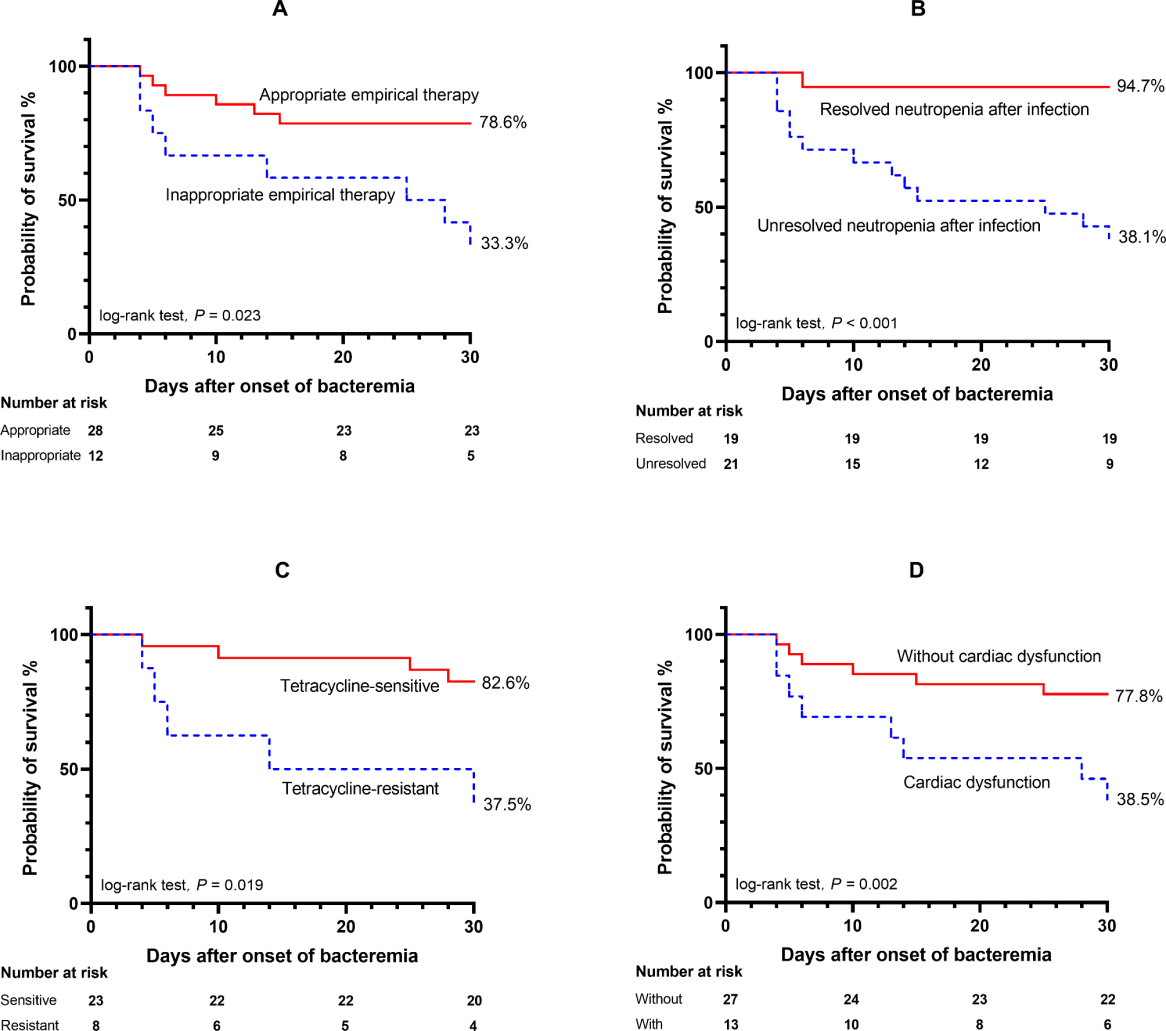
Kaplan-Meier curves of the 30-day probability of survival for patients with and without **(A)** appropriate empirical therapy, **(B)** resolved neutropenia after infection, **(C)** tetracycline-resistant *Acinetobacter* spp. BSI, and **(D)** cardiac dysfunction

The survival analysis showed that the 30-day survival probability of patients who received appropriate empirical antibiotic therapy was significantly higher than that of patients who received inappropriate empirical antibiotic therapy (78.6% [95% CI: 58.4–89.8%] vs. 33.3% [95% CI: 10.3–58.8%], P = 0.023) **(**Fig. 2A**)**. Unresolved neutropenia after infection (38.1% [95% CI: 18.3–57.8%] vs. 94.7% [95% CI: 68.1–99.2%], P < 0.001) and tetracycline-resistant *Acinetobacter* spp. BSI (37.5% [95% CI: 8.7–67.4%] vs. 82.6% [95% CI: 60.1–93.1%], P = 0.019) were associated with worse survival **(**Fig. 2B **and C)**. Furthermore, the 30-day survival probability of patients who experienced cardiac dysfunction was poorer than that of patients without cardiac dysfunction (38.5% [95% CI: 14.1–62.8%] vs. 77.8% [95% CI: 57.1–89.3%], P = 0.002) **(**Fig. 2D**)**.

### Strain identification

**Fig. 3:**
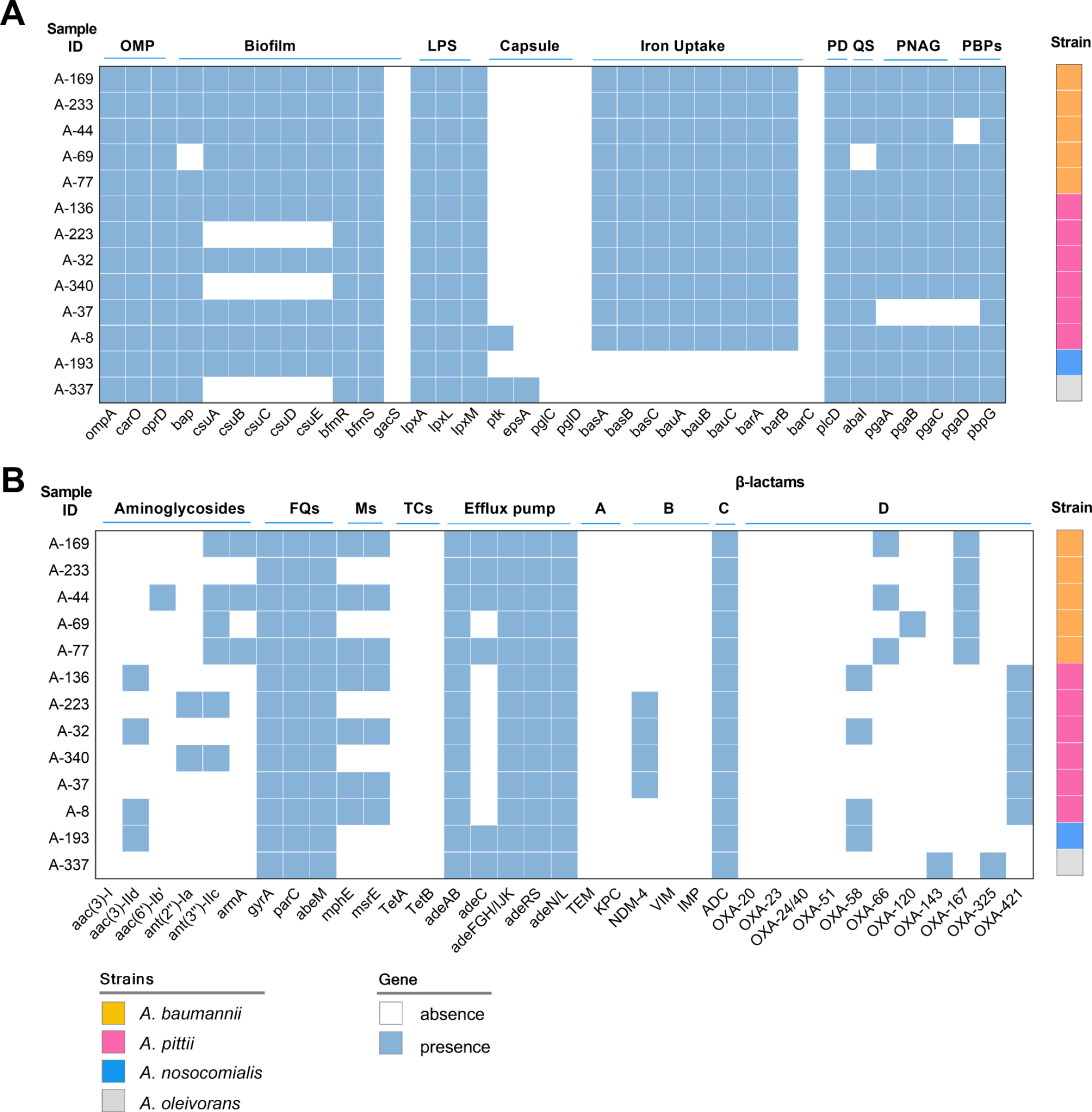
The vital virulence factors **(A)** and drug-resistance genes **(B)** of the remaining *Acinetobacter* strains isolated from the bloodstream. The text above represents the types of genes, and the text below represents the names of genes. Blue squares indicate gene positives, while white squares indicate gene negatives. OMP, outer membrane proteins; LPS, lipopolysaccharide; PD, phospholipase D; QS, quorum-sensing system; PNAG, beta-1-6-linked poly-N-acetyl glucosamine; PBPs, penicillin-binding proteins; FQs, fluoroquinolones; Ms, macrolides; TCs, tetracyclines; A, ambler class A of β-lactams antibiotics

This study used the VITEK 2 Compact to identify strains, most classified as *A. baumannii* or *A. calcoaceticus-baumannii* complex (Supplementary Table 3). Thirteen frozen CRA strains isolated from the bloodstream were resuscitated and identified, including *A. pittii* (n = 6), *A. baumannii* (n = 5), *A. calcoaceticus* (n = 1), and *A. nosocomialis* (n = 1) using matrix-assisted laser desorption/ionization time-of-flight mass spectrometry (MALDI-TOF MS). However, the WGS results reclassified the strain of *A. calcoaceticus* as *A. oleivorans* according to the GTDB. To our knowledge, this is the first report of *A.oleivorans* as a human pathogen. A 27-year-old patient experienced *A. oleivorans* bacteremia at the outset of treatment. The patient had been diagnosed with VSAA and and had a history of residing in a harbor development zone. Approximately 10 days before admission, the patient developed a fever, which was further complicated by respiratory tract and lower extremity soft tissue infections. This MDR isolate was sensitive to cefoperazone-sulbactam, fluoroquinolones, and tetracycline and resistant to carbapenems (Supplementary Table 2). The patient was eventually cured with ceftazidime-avibactam and tigecycline. The virulence and drug resistance genes of the *A. oleivorans* strain are shown in Fig. 3. This study found that the *A. oleivorans* strain did not carry common oxacillinases, such as OXA-23, OXA-24/40, OXA-51, OXA-58, and OXA-143. Instead, it carried a unique oxacillinase, OXA-325, which has not been previously reported in *A. oleivorans*.

## Discussion

Few studies have focused on *Acinetobacter* spp. BSI in hematological patients [14, 15]. Shargian-Alon et al. [14] reported a 7-day all-cause mortality rate of up to 72% in 46 patients with hematological malignancies who experienced an episode of CR *A. baumannii* bacteremia. A multicenter analysis in China [15], including 40 episodes of *A. baumannii* BSI in patients with hematological malignancy, obtained a mortality rate of 32.5%. In this study, the 30-day all-cause mortality rate in hematological patients with *Acinetobacter* spp. BSI was 35%, posing a serious threat to their prognosis. Therefore, clinicians must summarise the available evidence to take appropriate preventive measures and make effective therapeutic decisions.

This study reviewed cases of *Acinetobacter* spp. BSI in a large Chinese blood disease hospital over the last decade, and due to the low incidence, only 40 patients were enrolled. These patients did not show a significant concentration of age and gender distribution. Hematological patients with *Acinetobacter* spp. BSI presented with more severe conditions, with 22.5% of them developing respiratory failure (n = 9) and 25.0% developing severe sepsis or septic shock (n = 10). Additionally, they were more likely to have co-infections at multiple sites, including pneumonia, oral mucositis or pharyngitis, perianal infection, and skin or soft tissue infection. It is noteworthy that 12.5% of the patients suffered from intracranial infections, which differs from those reported with *Aeromonas* bacteremia [16], MDR *Pseudomonas aeruginosa* bacteremia [17], and CR *Enterobacteriaceae* bacteremia [13] in our centre.

Limited antibiotic drugs can be administered to patients with *Acinetobacter* spp. BSI owing to their high antibiotic resistance. It has been reported that inappropriate initial antibiotic treatment is administered to 88% of patients with *Acinetobacter* spp. BSI [21]. Consistent with several retrospective studies [22–24], the present study suggested that 30.0% of the patients received inappropriate empirical antibiotics, which was associated with adverse outcomes with a mortality rate of up to 66.7%. Additionally, some studies have recommended antibiotic combinations [12, 25, 26]. Tetracycline susceptibility rates reached 90% among carbapenem-resistant *Acinetobacter spp* [27], so Tetracycline-resistant *Acinetobacter* strains tended to be XDR. Consequently, *Acinetobacter* strains that exhibited resistance to tetracycline tended to be extensively drug-resistant (XDR). Our findings demonstrated that both XDRA and tetracycline-resistant *Acinetobacter* spp. were predictive of unfavorable prognoses. For XDR *Acinetobacter baumannii*, the Chinese guidelines [28] recommended several combination regimens, including those based on sulbactam and its combinations, tigecycline-based regimens, and polymyxin-based regimens. Owing to increasing resistance to carbapenems, colistin plus tigecycline and carbapenem plus tigecycline have become the most frequently used combinations [29]. Notably, a retrospective study that included 214 patients with XDR *A. baumannii* BSI showed no difference in hospital survival between patients receiving colistin monotherapy and those receiving combination therapy based on colistin [30]. Our data also showed that definitive combination therapy did not reduce 30-day mortality, which may be associated with the suboptimal dosing of colistin and the presence of heteroresistant strains. Furthermore, long-term hospital stays, invasive procedures, immunosuppressive therapy, and severe underlying illnesses have been reported to be risk factors for mortality^[22–24,31−33]^. The present study also demonstrated that more attention should be paid to improving neutropenia after infection and severe organ dysfunction to control the progression of bacteremia.

Notably, the resistance rate of *Acinetobacter* spp. to carbapenems is increasing, especially in the ICU, where up to 88% of *A. baumannii* isolates associated with hospital-acquired infections are CR [31]. It was controversial whether CRA acquisition was a poor prognostic factor [15, 32–34]. In this study, CRA BSI accounted for 40% of all cases. Consistent with previous studies [15, 35], the proportion of MDRA strains was higher than CRA (47.5% vs. 40.0%). The risk factors for CR *A. baumannii* BSI in previous reports included a history of carbapenem use, previous invasive procedures, length of hospital stay before bacteremia, hematological malignancies, and low socioeconomic status [32, 33, 36]. The present study showed that antimicrobial exposure at the onset of BSI and previous exposure to carbapenems within 30 days were associated with a significantly higher risk of CRA BSI. Additionally, some studies have reported a direct relationship between colonization pressure and infection acquisition in the intensive care unit [37–39]. The present study confirmed that CRA colonization was an independent risk factor for CRA BSI. We also found that patients with CRA bacteremia had worse clinical conditions before bacteremia, such as hypoproteinaemia and cardiac dysfunction, which may have affected their immunity and led to the invasion of CRA isolates. Therefore, avoiding the overuse of antibiotics is important for reducing drug resistance, and assessing the antibiotic susceptibility of colonized bacteria can provide early clues for the acquisition of CRA BSI.

The role of non-*A. baumannii*, such as *A. pittii* and *A. nosocomialis* in hospital-acquired infections, has been increasingly recognised. Compared with conventional methods of strain identification, MALDI-TOF MS has a nearly 100% identification rate for various bacteria [40]. The present study used three methods, the VITEK automated microbiology system, MALDI-TOF MS, and WGS, to identify *Acinetobacter*. These results illustrated the limitations of *Acinetobacter* spp. identification in clinical studies. In the present study, we first identified *A.oleivorans* as a human pathogen based on GTDB. It is an oil-degrading bacterium, mainly studied in environmental engineering. It was previously considered non-pathogenic [41]. The identification of the *A.oleivorans* strain suggests that an environment-originated non-pathogenic species can become pathogenic when the body’s immunity is compromised. In addition, the major oxacillinases (OXA-23, OXA-24/40, OXA-51, OXA-58, and OXA-143) are responsible for most carbapenem resistance detected in the United States, Europe, Asia, and other parts of the world [42]. This pathogen carries a unique oxacillinase, OXA-325, which was not previously reported in *A. oleivorans*.

The major limitation of this study was its small sample size, which may have affected the generalizability of the results. Moreover, the study’s retrospective nature made patient inclusion dependent on the physician’s clinical judgment, resulting in an inability to assess the patients comprehensively. On the other hand, we have compensated for the limitations of microbial identification in previous studies focusing on *A. baumannii* by identifying the genomospecies of *Acinetobacter* spp. using gene sequencing.

In conclusion, this study is the first to report *A. oleivorans* as a human pathogen. We found that hematological patients with *Acinetobacter* spp. BSI were more likely to be co-infected at multiple sites and have more severe conditions, which differed from those with other bacteremias at our center. This study demonstrated that prior administration of carbapenems, antimicrobial exposure at the onset of BSI, and CRA colonization increased the risk of subsequent CRA BSI. Tetracycline-resistant *Acinetobacter* spp. BSI and VSAA significantly affected the prognosis. Additionally, appropriate therapeutic decisions to reduce mortality include control of neutropenia, appropriate empirical antibiotic therapy, and timely improvement of severe complications.

### Electronic supplementary material

Below is the link to the electronic supplementary material.

Supplementary Material 1

## Data Availability

The datasets generated and/or analyzed during the current study are available in the GenBank repository. You can access them through the following web link: https://www.ncbi.nlm.nih.gov/nuccore/?term=PRJNA883531, and the accession number is **PRJNA883531**.
