# Supplementary material for: Acinetobacter spp. bloodstream infection in hematological patients: a 10-year single-center study
Source: BMC Infect Dis. 2023 Nov 14;23:796. doi: 10.1186/s12879-023-08789-6 (PMC10648370; doi:10.1186/s12879-023-08789-6)
Supplement: Supplementary file 1 — Supplementary Material 1 [file 12879_2023_8789_MOESM1_ESM.docx]

**Supplementary Data**

| **Table S1. The appropriateness of empirical treatment and the combination of definitive therapy.** | | | | | | |
| --- | --- | --- | --- | --- | --- | --- |
|  | **Inappropriate empirical treatment n=12** | **Appropriate empirical treatment n=28** | **P value** | **Definitive monotherapy**  **n=19** | **Definitive combination** **therapy**  **n=21** | **P value** |
| Carbapenem-resistant *Acinetobacter* spp. | 11 (91.7) | 5 (17.9) | ***<0.001**** | 3 (15.8) | 13 (61.9) | ***0.003**** |
| Multidrug-resistant *Acinetobacter* spp. | 12 (100.0) | 7 (25.0) | ***<0.001**** | 5 (26.3) | 14 (66.7) | ***0.011**** |
| Unresolved neutropenia after infection | 10 (83.3) | 11 (39.3) | ***0.011**** | 7 (36.8) | 14 (66.7) | ***0.059*** |
| Time of antibiotic treatment after BSI | 13 (4-44) | 14 (6-24) | 0.882 | 11 (6-15) | 21 (4-36) | 0.322 |
| Total length of hospital stay | 35 (22-59) | 35 (24-67) | 0.873 | 28 (23-43) | 37 (32-98) | ***0.031**** |
| 30-day microbial cure rate | 1 (8.3) | 16 (57.1) | ***0.004**** | 12 (63.2) | 5 (23.8) | ***0.012**** |
| 30-day mortality | 8 (66.7) | 6 (21.4) | ***0.011**** | 6 (31.6) | 8 (38.1) | 0.666 |
| Categorical variables are presented as numbers (percentiles); continuous variables are presented as median (interquartile range). BSI, bloodstream infection. | | | | | | |

| **Table S2. Drug resistance mode** **of all Acinetobacter strains isolated from bloodstream.** | | | | | | | | | | | | | | | | | | | | | | |
| --- | --- | --- | --- | --- | --- | --- | --- | --- | --- | --- | --- | --- | --- | --- | --- | --- | --- | --- | --- | --- | --- | --- |
| **ID** | ATM | AMC | TZP | CSL | MEM | IPM | DOR | CTX | CAZ | CRO | FEP | CIP | NOR | LVX | MFX | TOB | DOX | TCY | TGC | MNO | SXT | CST |
| **1** | 16 |  | <4 |  |  | <1 |  |  | 8 | 16 | 4 | <0.25 |  | <0.25 |  | <1 |  |  |  |  | <20 |  |
| **2** | >64 |  | >128 |  |  | >16 |  |  | >64 | >64 | >64 | >4 |  | >8 |  | >16 |  |  |  |  | <20 |  |
| **3** | 32 |  | <4 |  |  | <1 |  |  | 4 | 16 | 4 | <0.25 |  | <0.25 |  | <1 |  |  |  |  | <20 |  |
| **4** | 32 |  | <4 |  |  | <1 |  |  | 8 | 16 | 8 | <0.25 |  | <0.25 |  | <1 |  |  |  |  | <20 |  |
| **5** | 32 |  | <4 |  |  | <1 |  |  | 16 | 16 | 8 | <0.25 |  | <0.25 |  | <1 |  |  |  |  | <20 |  |
| **6** | 16 |  | <4 |  |  | <1 |  |  | 4 | 16 | 2 | <0.25 |  | <0.25 |  | <1 |  |  |  |  | <20 |  |
| **7** | 4 |  | <4 |  |  | <1 |  |  | 2 | 8 | <1 | <0.25 |  | <0.25 |  | <1 |  |  |  |  | <20 |  |
| **8** | 32 |  | <4 |  |  | <1 |  |  | 4 | 16 | 2 | <0.25 |  | <0.25 |  | <1 |  |  |  |  | <20 |  |
| **9** | >64 |  | <4 |  |  | <1 |  |  | 8 | 16 | 4 | <0.25 |  | <0.25 |  | <1 |  |  |  |  | <20 |  |
| **10** | >64 | >32 | >128 |  |  | >16 | >8 | >64 | 16 | >64 | >64 | >4 | >16 | 4 | 1 | >16 |  | >16 | >8 |  | 160 |  |
| **11** | >64 | 16 |  |  |  | >16 | >8 | >64 | 4 | >64 | >64 | 1 | 4 | 2 | <0.25 | >16 |  | >16 | 2 |  | <20 |  |
| **12** | 16 | 4 | 8 |  |  | <0.25 | <0.12 | 8 | 4 | 16 | 2 | <0.25 | 2 | <0.12 | <0.25 | <1 |  | <1 | <0.5 |  | <20 |  |
| **13** | >64 | >32 | >128 |  |  | 8 | >8 | 8 | 32 | <1 | 4 | <0.25 | 2 | 1 | <0.25 | >16 |  | 4 | 1 |  | <20 |  |
| **14** | >64 | 4 | <4 |  |  | <0.25 | 0.25 | 16 | 4 | 16 | 4 | <0.25 | 2 | 0.25 | <0.25 | <1 |  | <1 | <0.5 |  | <20 |  |
| **15** | >64 | 16 | >128 |  | 0.5 | <0.25 | 0.25 | >64 | 16 | 32 | 32 | <0.25 | 2 | <0.12 | <0.25 | <1 |  | <1 | <0.5 |  |  |  |
| **16** | 16 | >32 | >128 |  | >16 | >16 | >8 | 16 | 16 | 16 | 4 | >4 | >16 | >8 | >8 | >16 |  | >16 | >8 |  | >320 |  |
| **17** | >64 | >32 | >128 |  | 8 | >16 | >8 | 32 | 16 | 32 | >64 | >4 | >16 | >8 | >8 | <1 |  | 2 | <0.5 |  |  |  |
| **18** | 32 | 4 | 8 |  | <0.25 | <0.25 | <0.12 | 16 | 4 | 16 | 2 | <0.25 | 2 | <0.12 | <0.25 | <1 |  | <1 | <0.5 |  | <20 |  |
| **19** | 32 | >32 | >128 |  | >16 | >16 | >8 | >64 | >64 | >64 | >64 | >4 | >16 | >8 | >8 | >16 |  | 4 | 2 |  | >320 |  |
| **20** | >64 | 16 | >128 |  | >16 | >16 | >8 | >64 | >64 | >64 | >64 | <0.25 | 2 | <0.12 | <0.25 | <1 |  | <1 | <0.5 |  | >320 |  |
| **21** | 32 | 8 | <4 |  | <0.25 | <0.25 | <0.12 | 8 | 4 | 16 | 2 | 0.5 | 2 | <0.12 |  | <1 |  | <1 | <0.5 |  | <20 |  |
| **22** | >64 | 16 | >128 |  | 8 | 8 | >8 | >64 | >64 | >64 | >64 | >4 | >16 | >8 | >8 | <1 |  | 4 | <0.5 |  | <20 |  |
| **23** | 32 | 8 | <4 |  | <0.25 | <0.25 | <0.12 | 16 | 4 | 16 | 2 | <0.25 | 2 | <0.12 | <0.25 | <1 |  | <1 | <0.5 |  | <20 |  |
| **24** | 16 | 8 | <4 |  | <0.25 | <0.25 | <0.12 | 8 | 4 | 16 | 2 | <0.25 | 2 | <0.12 | <0.25 | <1 |  | <1 | <0.5 |  | <20 |  |
| **25** | 32 | 16 | <4 |  | <0.25 | <0.25 | <0.12 | 16 | 8 | 16 | 8 | <0.25 | 2 | <0.12 | <0.25 | <1 |  | <1 | <0.5 |  | <20 |  |
| **26** | >64 | >32 | >128 |  | >16 | >16 | >8 | >64 | >64 | >64 | >64 | >4 | >16 | >8 | >8 | >16 |  | >16 | 2 |  | <20 |  |
| **27** | >64 | 8 | 8 |  | <0.25 | <0.25 | <0.12 | 8 | 16 | 16 | 4 | <0.25 | 2 | <0.12 | <0.25 | <1 |  | <1 | <0.5 |  | <20 |  |
| **28** | >64 | >32 | >128 |  | >16 | >16 | >8 | >64 | >64 | >64 | >64 | >4 | >16 | >8 | >8 | 4 |  | >16 | 2 |  | <20 |  |
| **29** | >64 | >32 | >128 |  | 8 | >16 | >8 | 32 | 16 | 16 | 32 | >4 | >16 | >8 | >8 | <1 |  | <1 | <0.5 |  | 160 |  |
| **30** | 32 | 4 | <4 |  | <0.25 | <0.25 | <0.12 | 16 | 4 | 16 | 2 | <0.25 | 2 | <0.12 | <0.25 | <1 |  | <1 | <0.5 |  | <20 |  |
| **31** | 32 | 8 | 8 |  | <0.25 | <0.25 | 0.25 | 16 | 8 | 16 | 4 | <0.25 | 2 | <0.12 | <0.25 | <1 |  | <1 | <0.5 |  | <20 |  |
| **32** | 16 | 8 | <4 |  | 1 | <0.25 | 1 | 8 | 8 | 16 | 32 | 1 | 8 | 1 | 0.5 | >16 |  | 2 | 4 |  | >320 |  |
| **33** | 4 | 4 | <4 |  | <0.25 | <0.25 | <0.12 | 4 | 2 | 4 | <1 | <0.25 | 2 | <0.12 | <0.25 | <1 |  | <1 | <0.5 |  | <20 |  |
| **34** | >64 | >32 | >128 |  | >16 | >16 | >8 | >64 | >64 | >64 | >64 | >4 | >16 | >8 | 4 | >16 |  | 2 | <0.5 |  | <20 |  |
| **35** | >64 | >32 | >128 |  | >16 | >16 | >8 | >64 | 32 | 32 | >64 | 2 | >16 | 0.5 | <0.25 | <1 |  | 2 | 2 |  | <20 |  |
| **36** | 16 | 4 | 8 |  | <0.25 | <0.25 | 0.5 | 8 | 8 | 16 | <1 | <0.25 | 2 | <0.12 | <0.25 | <1 |  | >16 | <0.5 |  | <20 |  |
| **37** | 16 | 4 | 16 | <8 | <0.25 | <0.25 | 0.25 | 8 | 2 |  | 2 | <0.25 | 2 | <0.12 | <0.25 | <1 |  | <1 | <0.5 |  | <20 |  |
| **38** | 16 | 8 | >128 | 16 | 8 | 8 | >8 | >64 | >64 |  | >32 | >4 | >16 | 4 | 1 | >16 |  | 4 | <0.5 |  | <20 |  |
| **39** | 32 | 8 | <4 | <8 | <0.25 | <0.25 | <0.12 | 8 | 2 |  | 2 | <0.25 | 2 | <0.12 | <0.25 | <1 |  | <1 | <0.5 |  | <20 |  |
| **40** | ≥64 | ≥32 | ≥128 | ≤8 | ≥16 | 8 | ≥8 | 32 | 16 | 32 | 8 | ≤0.25 | 2 | ≤0.12 | ≤0.25 | ≤1 | ≤0.5 | 2 | ≤0.5 | ≤1 | ≤20 | ≤0.5 |
| ATM, aztreonam; AMC, amoxicillin-clavulanic acid; TZP, piperacillin-tazobactam; CSL, cefoperazone-sulbactam; MEM, meropenem; IPM, imipenem; DOR, doripenem; CTX, cefotaxime; CAZ, ceftazidime; CRO, ceftriaxone; FEP, cefepime; CIP, ciprofloxacin; NOR, norfloxacin; LVX, levofloxacin; MFX, moxifloxacin; TOB, tobramycin; DOX, doxycycline; TCY, tetracycline; TGC, tigecycline；MNO, minocycline; SXT, trimethoprim-sulfamethoxazole; CST, colistin. | | | | | | | | | | | | | | | | | | | | | | |

| **Table S3. Comparison of** **strain identification results using different methods.** | | | | |
| --- | --- | --- | --- | --- |
|  |  | **Identification methods** | | |
| **ID** | **Origin** | **VITEK 2 Compact** | **MALDI-TOF MS** | **WGS** |
| **A-69** | bloodstream | *A. baumannii* | *A. baumannii* | *A. baumannii* |
| **A-77** |  |  |  |  |
| **A-193** |  |  | *A. nosocomialis* | *A. nosocomialis* |
| **A-8** |  |  | *A. pittii* | *A. pittii* |
| **A-37** |  |  |  |  |
| **A-44** |  | *A. calcoaceticus-baumannii c*omplex | *A. baumannii* | *A. baumannii* |
| **A-169** |  |  |  |  |
| **A-233** |  |  |  |  |
| **A-337** |  |  | *A. calcoaceticus* | *A. oleivorans* |
| **A-32** |  |  | *A. pittii* | *A. pittii* |
| **A-136** |  |  |  |  |
| **A-223** |  |  |  |  |
| **A-340** |  |  |  |  |
| ID represents the number of the strain sample. MALDI-TOF MS, matrix-assisted laser desorption/ionization-time-of-flight mass spectrometry; WGS, whole genome sequencing. | | | | |
